# Tertiary lymphoid structures combined with biomarkers of inflammation are associated with the efficacy of neoadjuvant immunochemotherapy in resectable non‐small cell lung cancer: A retrospective study

**DOI:** 10.1111/1759-7714.15175

**Published:** 2023-12-06

**Authors:** Fuhao Xu, He Zhu, Dali Xiong, Kang Wang, Yinjun Dong, Li Li, Shuanghu Yuan

**Affiliations:** ^1^ Department of Radiation Oncology Shandong Cancer Hospital and Institute, Shandong First Medical University and Shandong Academy of Medical Sciences, Shandong Cancer Hospital Affiliated to Shandong First Medical University Jinan China; ^2^ Department of Thoracic Surgery Shandong Cancer Hospital and Institute, Shandong First Medical University and Shandong Academy of Medical Sciences Jinan China

**Keywords:** biomarkers, immune checkpoint inhibitor, neoadjuvant therapy, non‐small‐cell lung cancer, tertiary lymphoid structure

## Abstract

**Background:**

Neoadjuvant immunochemotherapy can effectively downstage tumors and reduce the risk of postoperative recurrence and distant metastasis in patients with non‐small cell lung cancer (NSCLC). In this study, we investigated the correlation between inflammatory biomarkers and tertiary lymphoid structure (TLS) expression. We also compared the predictive values of these inflammatory parameters, TLSs, and a combination of inflammatory parameters and TLSs for neoadjuvant efficacy in patients with NSCLC.

**Methods:**

We retrospectively analyzed the clinical information of 106 patients with NSCLC who underwent neoadjuvant immunochemotherapy and radical surgery at Shandong Cancer Hospital between June 2020 and June 2022.

**Results:**

TLS was evaluated using hematoxylin–eosin staining and immunohistochemically‐stained tissue sections. Logistic analysis was performed to determine the correlation between inflammatory parameters, TLSs, and the factors affecting major pathological response (MPR). Receiver operating characteristic curves and the C‐index were used to evaluate the predictive value of the nomogram models for MPR. The systemic immune‐inflammatory index (SII) was an independent predictor of high TLS abundance and maturity. Platelet‐to‐lymphocyte ratio (PLR) ≤201.8, TLS abundance, and TLS maturity were independent predictors of MPR. The PLR‐TLS combined model performed better in assessing the MPR in patients with NSCLC than models using single indicators.

**Conclusion:**

Our study demonstrated that the SII is an independent predictor of both TLS abundance and maturity. Both TLSs and PLR can predict MPR rates in patients with NSCLC receiving neoadjuvant immunochemotherapy. However, assessing the MPR in patients with NSCLC using a combination of PLR and TLSs is more accurate than using either indicator alone.

## Graphical abstract

This retrospective study of 106 patients with NSCLC receiving neoadjuvant immunochemotherapy identified the SII, a systemic inflammation index, as an independent factor influencing TLS abundance and maturity, with a higher SII negatively associated with TLS expression in the tumor microenvironment. Moreover, compared with using TLSs or PLR individually, combining them more accurately predicted major pathological response rates to neoadjuvant immunochemotherapy.

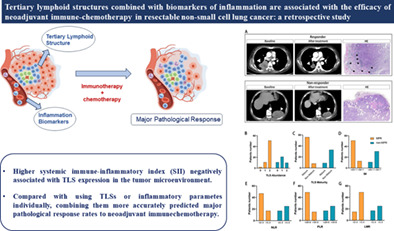

## INTRODUCTION

Surgical resection remains the primary treatment for non‐small cell lung cancer (NSCLC), but some patients still experience distant metastasis and recurrence postoperatively. This poor outcome may be attributed to the presence of residual micrometastases or subclinical lesions.^1^, ^2^ In many patients with NSCLC, local therapies alone are insufficient to prevent recurrence, underscoring the need for perioperative treatments to improve NSCLC outcomes. Neoadjuvant chemotherapy administered before definitive local radiotherapy or surgery aims to shrink primary tumors, eradicate subclinical lesions, facilitate subsequent therapies, and improve patient prognosis. Although traditional neoadjuvant chemotherapy can improve prognosis in patients with NSCLC, the 5‐year absolute survival rate only increases by 5%, suggesting that there is still room for improvement in neoadjuvant therapy in patients with initially resectable NSCLC.^3^, ^4^

In recent years, immune checkpoint inhibitors (ICIs) targeting programmed cell death receptor 1 (PD‐1) and its ligand, PD‐L1, have demonstrated better safety and efficacy than chemotherapy for advanced driver mutation‐negative NSCLC.^5^, ^6^, ^7^ The application of ICIs in neoadjuvant therapy for initially resectable NSCLC can effectively downstage tumors and reduce the risk of postoperative recurrence and distant metastasis, resulting in a significantly improved prognosis.^8^, ^9^, ^10^ The results from the phase 3 CheckMate‐816 trial showed that compared to surgery alone, preoperative application of 2–4 cycles of nivolumab plus chemotherapy could improve long‐term survival, with the 3‐year disease‐free survival rate significantly increasing from 50% to 63%.^9^ The majority of evidence of the advantages of neoadjuvant immune therapy comes from phase I/II clinical trials, which have shown major pathological response (MPR) rates ranging from 19% to 45% for single‐agent immunotherapy and from 33% to 83% for immunotherapy combined with other neoadjuvant therapies.^11^, ^12^, ^13^ However, not all patients benefit from neoadjuvant immunochemotherapy, and the degree of benefit varies among individuals. For patients insensitive to neoadjuvant immunochemotherapy, neoadjuvant therapy not only delays the best treatment timing but also incurs high treatment costs.^10^ Therefore, effectively identifying the beneficiaries of neoadjuvant immunochemotherapy in NSCLC and enabling the effective monitoring and prediction of neoadjuvant treatment responses are of critical importance.

Tertiary lymphoid structures (TLSs) are organized aggregates of immune cells, including B cells, T cells, and dendritic cells, which arise in nonlymphoid tissues.^14^ Accumulating evidence indicates that adaptive immune responses can be initiated or enhanced within TLSs, and that certain antitumor antibodies are closely associated with B cells in TLSs.^15^ Ng et al. found that B cells and antibody responses in TLSs can target endogenous retroviral envelope proteins, which can be enhanced by immunotherapy.^16^ In hepatocellular carcinoma research, scientists have found that an intra‐tumoral cellular triad of PD‐1^hi^CD8^+^ T cells, CXCL13^+^ T helper cells, and mature regulatory DCs rich in modulatory molecules promote immune responses induced by PD‐1 inhibitor therapy.^17^ The high abundance and maturity of TLSs have been validated as favorable diagnostic factors for immunotherapy in various tumors including hepatocellular carcinoma,^18^ melanoma,^19^ and sarcoma.^20^

TLSs form under chronic inflammatory stimuli. Their structure and function can be modulated by specific inflammatory cues, leading to variations across different tissues and diseases. Unlike lymph nodes, TLSs lack an outer encapsulating structure, which makes the inner immune cells more susceptible to constant inflammatory exposure.^21^ Cytokines and metabolic factors produced in the surrounding tumor tissues can directly contact immune cells in TLSs, thereby influencing immune responses.^22^, ^23^ Therefore, we believe that evaluating TLSs based solely on their abundance and maturity has limitations. Incorporating the inflammatory status of patients may enable a more comprehensive assessment. The systemic immune inflammatory index (SII) is based on a prognostic score of inflammation and immunity, which is calculated as platelet count*neutrophil count/lymphocyte count. Inflammation induces leukocytosis, and the systemic immune‐inflammatory index (SII), neutrophil‐to‐lymphocyte ratio (NLR), lymphocyte‐to‐monocyte ratio (LMR), and platelet‐to‐lymphocyte ratio (PLR) can serve as inflammatory biomarkers and have been validated as diagnostic predictors in patients with lung and liver cancers receiving immunotherapy.^24^, ^25^

Therefore, we investigated the effects of inflammatory biomarkers (SII, PLR, NLR, and LMR) on TLS abundance and maturity of TLSs. We also compared the predictive values of these inflammatory parameters, TLSs, and the combination of inflammatory parameters with TLSs for immunotherapy efficacy in patients with NSCLC receiving neoadjuvant immunochemotherapy.

## METHODS

### Patients and samples

**FIGURE 1:**
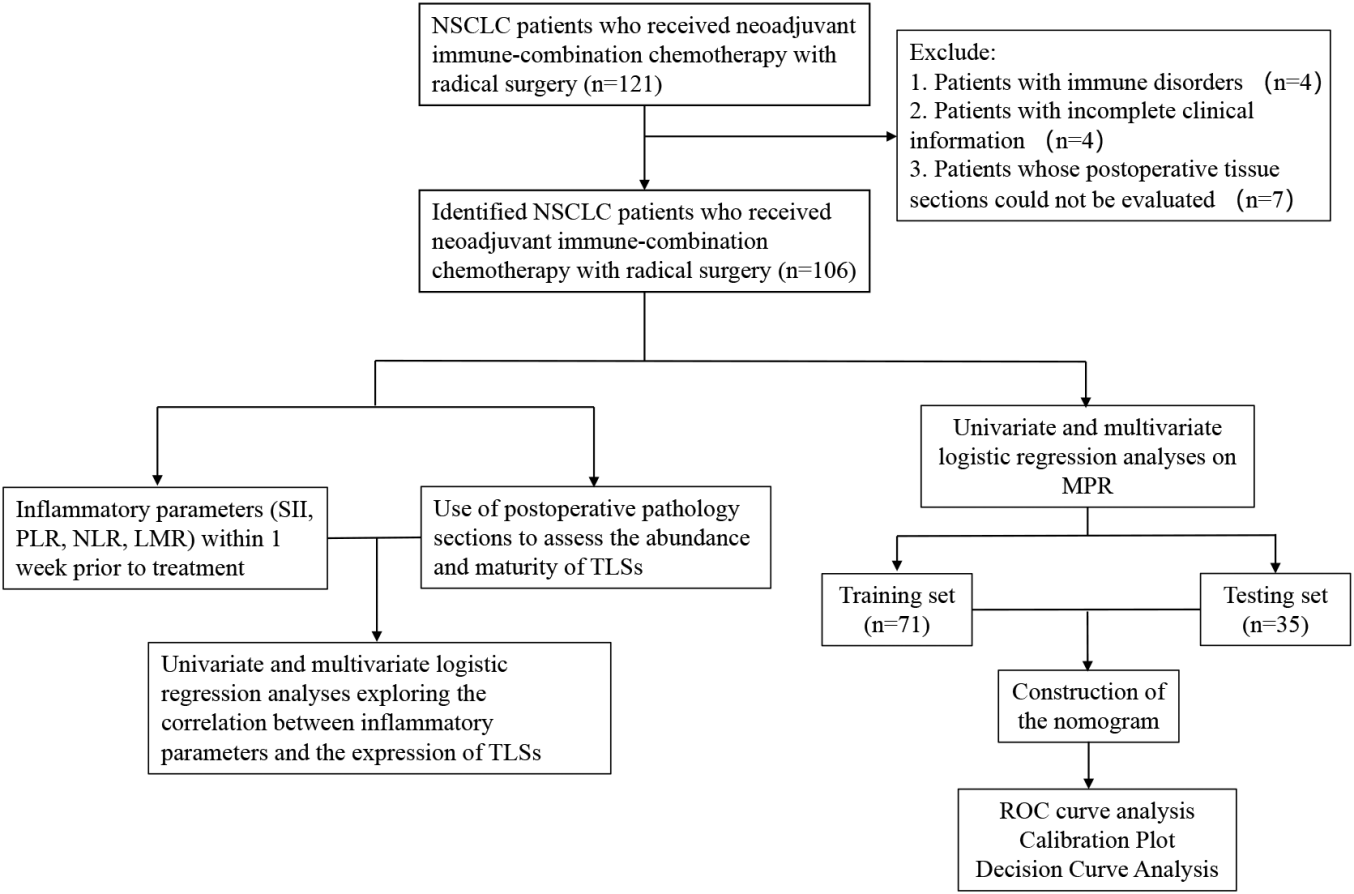
Flow chart for the inclusion and exclusion of non‐small cell lung cancer (NSCLC) patients and analysis of tumor information.

We retrospectively enrolled 106 patients with NSCLC pathologically diagnosed at Shandong Cancer Hospital between January 2020 and August 2022. Inclusion criteria were patients with predominantly resectable stage IB (≥4 cm) to IIIB with no prior antitumor therapy. All patients underwent radical surgery and received at least two cycles of PD‐1 inhibitors combined with taxanes and platinum‐based drugs preoperatively. Patients with active or prior medical history of immune‐related diseases were excluded. The inclusion and exclusion of patients with NSCLC and the tumor information analyses are shown in Figure 1.

### Data collection

For the enrolled patients with NSCLC, postoperative paraffin blocks, tumor information, postoperative pathology data, and routine blood tests within 1 week before neoadjuvant immunochemotherapy were collected. Key data included sex, age, pathology type, TNM stage, neutrophil count, monocyte count, lymphocyte count, platelet count, TLS abundance, and tissue section maturity. NSCLC staging was based on the AJCC eighth edition Cancer Staging System.

### Histopathological analysis

Two sections were prepared for each paraffin‐embedded tissue block. The sections were subjected to hematoxylin–eosin (HE) staining, and the abundance of TLSs in tumor tissues was scored as 0, 1, or 2: (A) 0 indicates no TLS, (B) 1 indicates <3 TLSs in the tumor, and (C) 2 indicates ≥3 TLSs in the tumor. Patients with a score of 2 were as assigned to the high‐abundance group; otherwise, patients were assigned to the low‐abundance group. According to previous studies, TLSs displaying active germinal centers (GCs) were defined as fully matured TLSs.^26^ We assessed the TLSs within the tumor and within the 5‐mm peritumoral area. The TLS scoring was validated by immunohistochemical staining. The TLS assessment was performed independently by two experienced pathologists in a blinded manner. In cases of disagreement between the two pathologists, the higher score was used.

### Statistical analysis

MPR was defined as the presence of ≤10% residual tumor cells in the surgical resection specimen, while the non‐MPR group included patients who did not achieve MPR. Statistical analyses were performed using the R Studio software (R Foundation for Statistical Computing) and SPSS (version 25.0; IBM Corporation). Receiver operating characteristic (ROC) curves were used to determine the optimal cutoff values for blood parameters (SII, PLR, LMR, and NLR). Univariate and multivariate binary logistic regression analyses were used to determine the correlations between inflammatory parameters and TLS abundance and maturity, as well as independent factors influencing MPR. *p* < 0.05 was considered statistically significant. Based on the identified independent predictors, patients were divided into training and validation cohorts in a 2:1 ratio. In addition, using R statistical software, nomogram models were constructed based on inflammatory biomarkers, TLS abundance and maturity, and their combinations to predict MPR rates. Furthermore, ROC curve analysis and the C‐index were used to compare the performances of the three models.

## RESULTS

### Patient characteristics

**FIGURE 2:**
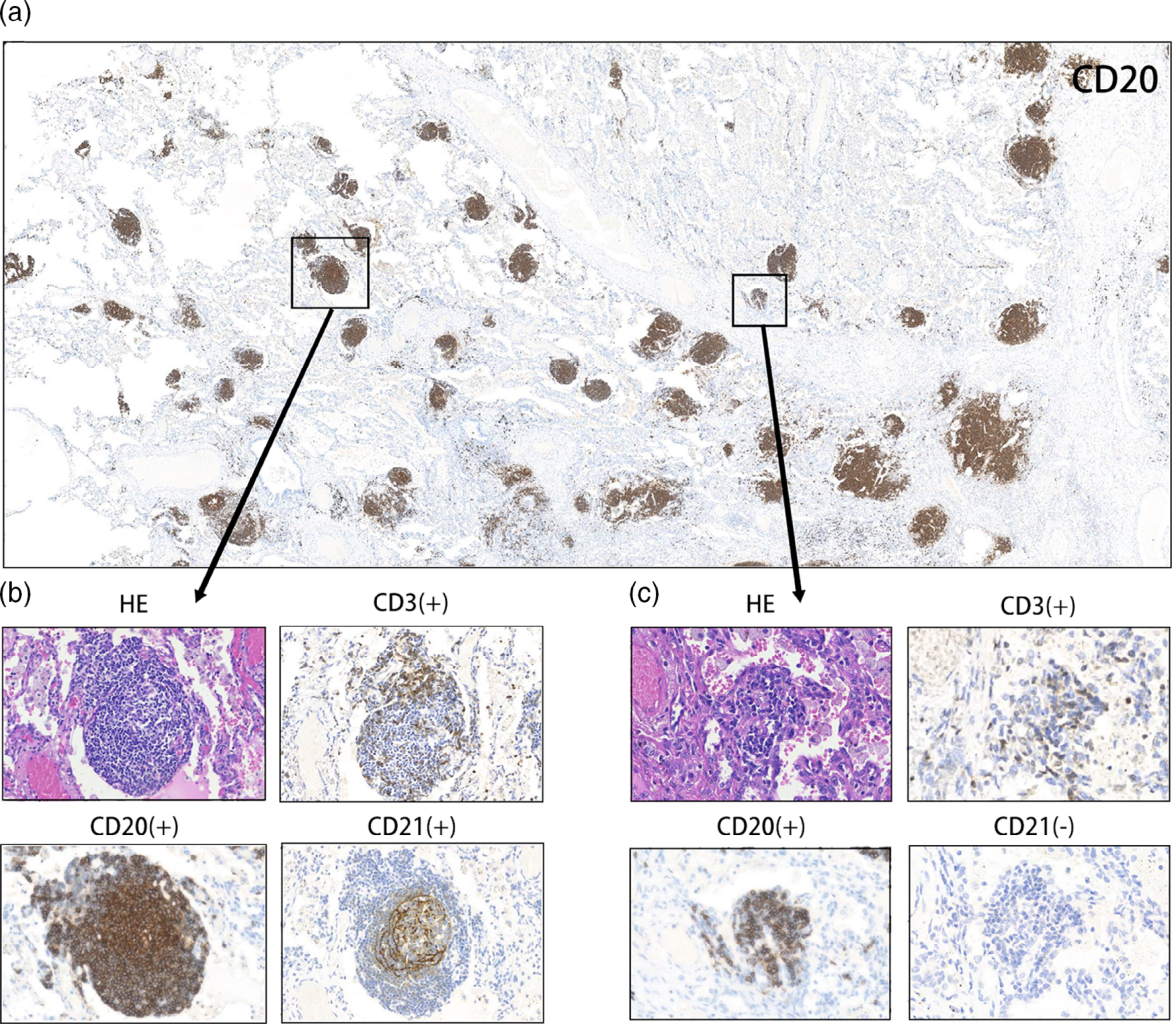
Different levels of maturity of the tertiary lymphoid structures. (a) Representative image of CD20 staining of tertiary lymphoid structures (TLSs) in a responder after neoadjuvant treatment. (b) Mature tertiary lymphatic structure, dense lymphocytic aggregates (CD3+, CD20+) with follicular dendritic cells (CD21+); (c) Immature tertiary lymphatic structures, dense lymphocytic aggregates (CD3+, CD20+) without follicular dendritic cells (CD21−).

This study included 106 patients with NSCLC undergoing neoadjuvant immunochemotherapy followed by radical surgery, of whom 94 (88.7%) were male. The median age at diagnosis was 63 years (range 44–102 years). A total of 78 (73.6%) patients had squamous cell carcinoma and 28 (26.4%) had adenocarcinoma. MPR was achieved in 64 patients (60.4%) **(**Table 1 **)**. Among the enrolled patients, 13 (12.2%), 23 (21.7%), and 61 (57.5%) had TLS abundance scores of 0, 1, and 2, respectively. Mature TLSs were observed in 65 (61.3%) patients. The different maturity states of the TLSs are shown in Figure 2.

**TABLE 1:** Basic characteristics of patients.

Variable	MPR (*n* = 64)	Non‐MPR (*n* = 42)
Age		
<63	31 (48.4%)	21 (50.0%)
≥ 63	33 (51.6%)	21 (50.0%)
Gender		
Male	56 (87.5%)	38 (90.5%)
Female	8 (12.5%)	4 (9.5%)
Smoking history		
Smokers	38 (59.4%)	26 (61.9%)
Never smokers	26 (40.6%)	16 (38.1%)
Histology		
Squamous carcinoma	51 (79.7%)	27 (64.3%)
Adenocarcinoma	13 (20.3%)	15 (35.7%)
TNM stage		
I + II	21 (32.8%)	10 (23.8%)
III	43 (67.2%)	32 (76.2%)
SII		
≤857.7	51 (79.7%)	11 (26.2%)
>857.7	13 (20.3%)	31 (73.8%)
NLR		
≤3.5	47 (73.4%)	17 (40.5%)
>3.5	17 (26.6%)	25 (59.5%)
PLR		
≤201.8	49 (76.6%)	17 (40.5%)
>201.8	15 (23.4%)	25 (59.5%)
LMR		
≤2.5	15 (23.4%)	17 (40.5%)
>2.5	49 (76.6%)	25 (59.5%)
TLS density		
Score of 0	2 (3.1%)	11 (26.2%)
Score of 1	11 (17.2%)	21 (50.0%)
Score of 2	51 (79.7%)	10 (23.8%)
TLS maturity		
Mature	56 (87.5%)	9 (21.4%)
Nonmature	8 (12.5%)	33 (78.6%)

*Note*: Statistical significance was set at *p* < 0.05. The expected count should be less than 5 to follow the Fisher's exact test results.

Abbreviations: CI, confidence interval; LMR, lymphocyte‐to‐monocyte ratio; MPR, major pathological response; NLR, neutrophil‐to‐lymphocyte ratio; PLR, platelet‐to‐lymphocyte ratio; SII, systemic immune‐inflammatory index; TLS, tertiary lymphatic structure.

### Correlation of systemic inflammatory parameters with TLS abundance and maturation

**FIGURE 3:**
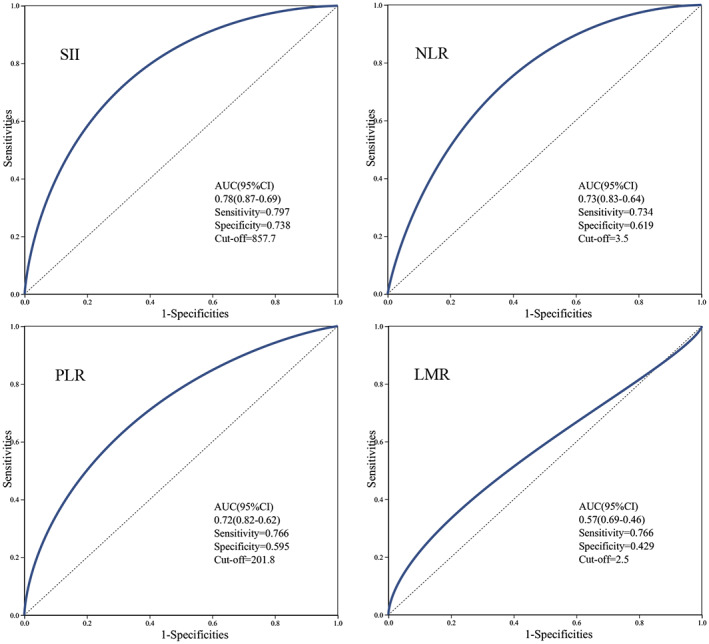
Receiver operating characteristic curve of inflammatory parameters based on the patient MPR. (a) The best cutoff for SII is 857.7, and when using this point for segmentation, the sensitivity is 0.797 and the specificity is 0.738. (b) The best cutoff for NLR is 3.5, and when using this point for segmentation, the sensitivity is 0.734 and the specificity is 0.619. (c) The best cutoff for PLR is 201.8, and when using this point for segmentation, the sensitivity is 0.766 and the specificity is 0.595. (d) The best cutoff for LMR is 2.5, and when using this point for segmentation, the sensitivity is 0.766 and the specificity is 0.429. LMR, lymphocyte‐to‐monocyte ratio; MPR, major pathological response; NLR, neutrophil‐to‐lymphocyte ratio; PLR, platelet‐to‐lymphocyte ratio; SII, systemic immune‐inflammatory index.

The optimal cutoff values for pretreatment SII, NLR, PLR, LMR, and lymphocyte count were determined using ROC curves and the maximum Youden index (Figure 3). Optimal cutoff values were used to categorize the inflammatory indices. TLS abundance and maturity were stratified to form two groups to investigate the relationship between inflammatory indices and TLSs in different patients with NSCLC. Univariate analysis revealed that SII (odds ratio [OR] = 0.258, *p* = 0.001), NLR (OR = 0.309, *p* = 0.005), and lymphocyte count (OR = 2.722, *p* = 0.024) correlated with TLS abundance. TNM stage (OR = 0.352, *p* = 0.032), SII (OR = 0.184, *p* < 0.05), and NLR (OR = 0.323, *p* = 0.007) were correlated with TLS maturity. Further multivariate logistic regression analysis found that SII was an independent predictor of both TLS abundance (OR = 0.273, 95% CI: 0.094–0.796, *p* = 0.017) and TLS maturity (OR = 0.227, 95% CI: 0.077–0.668, *p* = 0.007), and SII ≤857.7 was a protective factor against high TLS abundance and maturity (Supporting Information**)**. In summary, we found that SII was an independent influencing factor for TLS expression and that SII was negatively correlated with TLS expression status in the tumor microenvironment.

### Correlation between inflammatory parameters, TLSs, and pathological response to neoadjuvant immune combination chemotherapy in patients with NSCLC

**FIGURE 4:**
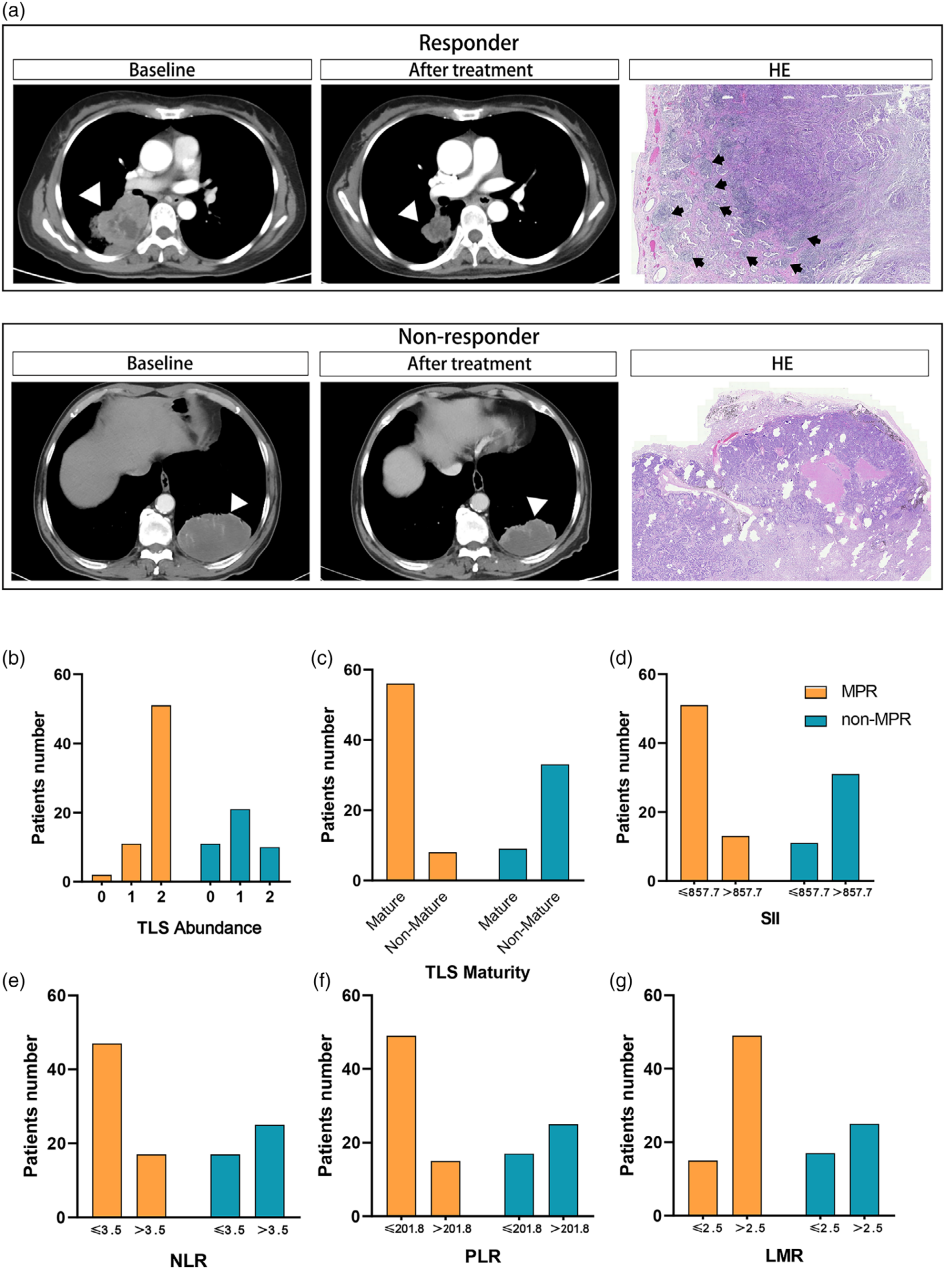
Comparison of TLS expression and inflammatory parameters in MPR and non‐MPR groups. (a) Comparison of CT and H&E images between responder and nonresponder before and after neoadjuvant therapy. Black arrows point to tertiary lymphoid structures. (b) Comparison of TLS abundance between MPR and non‐MPR groups. (c) Comparison of TLS maturity between MPR and non‐MPR groups. (d) Comparison of SII between MPR and non‐MPR groups. (e) Comparison of NLR between MPR and non‐MPR groups. (f) Comparison of PLR between MPR and non‐MPR groups. (g) Comparison of LMR between MPR and non‐MPR groups. LMR, lymphocyte‐to‐monocyte ratio; MPR, major pathological response; NLR, neutrophil‐to‐lymphocyte ratio; PLR, platelet‐to‐lymphocyte ratio; SII, systemic immune‐inflammatory index; TLS, tertiary lymphoid structure.

Comparison of CT and H&E images between responder and nonresponders before and after neoadjuvant therapy is shown in Figure 4a. Based on the pathological response after treatment, patients with NSCLC were divided into MPR and non‐MPR groups. Among the MPR patients, the numbers of cases with TLS abundance scores of 0, 1, and 2 were 2, 11, and 51, respectively, and among the non‐MPR patients, the numbers were 11, 21, and 10, respectively. The number of MPR patients with and without mature TLSs was 56 and eight, respectively; for non‐MPR patients, the numbers were nine and 33, respectively **(**Figure 4b,c **)**. A comparison of the inflammatory parameters between the MPR and non‐MPR groups is shown in Figure 4d–g. Univariate analysis revealed that the SII (OR = 0.090, *p* < 0.05), NLR (OR = 0.246, *p* = 0.001), PLR (OR = 0.208, *p* < 0.05), TLS abundance (*p* < 0.05), and TLS maturity (OR = 25.667, *p* < 0.05) correlated with patient MPR. Further multivariate logistic regression analysis of the above five factors identified PLR ≤201.8 (OR = 0.187, 95% CI: 0.036–0.965, *p* = 0.045), TLS abundance (*p* = 0.019), and TLS maturity = 1 (OR = 23.9737, 95% CI: 5.063–113.521, *p* < 0.05) as independent predictors of MPR **(**Table 2 **)**.

**TABLE 2:** Univariate and multivariate logistic analyses on MPR.

Variable	Univariate analysis		Multivariate analysis	
	*p*‐value	OR (95% CI)	*p*‐value	OR (95% CI)
Age	0.485	1.071 (0.971–1.065)		
Gender	0.637	0.737 (0.207–2.621)		
Male				
Female				
Smoking history	0.795	0.899 (0.405–1.998)		
Smokers				
Never smokers				
Histology	0.082	0.459 (0.191–1.103)		
Squamous carcinoma				
Adenocarcinoma				
TNM stage	0.321	0.640 (0.265–1.544)		
I + II				
III				
SII^a^	<0.05	0.090 (0.036‐0.227)	0.054	0.198 (0.038–1.030)
NLR^b^	0.001	0.246 (0.107‐0.564)	0.607	1.498 (0.321–7.000)
PLR^c^	<0.05	0.208 (0.089‐0.485)	0.045	0.187 (0.036–0.965)
LMR^d^	0.064	2.221 (0.954‐5.172)		
TLS density (vs. 0)	<0.05		0.019	
Score of 1	0.215	2.881 (0.540–15.365)	0.303	0.324 (0.038–2.767)
Score of 2	<0.05	28.050 (5.376–146.362)	0.420	2.386 (0.288–19.752)
TLS maturity	<0.05	25.667 (9.027–72.980)	<0.05	23.973 (5.063–113.521)
Mature				
Nonmature				

*Note*: Statistical significance was set at *p* < 0.05.

Abbreviation: CI, confidence interval; LMR, lymphocyte‐to‐monocyte ratio; MPR, major pathological response; NLR, neutrophil‐to‐lymphocyte ratio; OR, overall response: PLR, platelet‐to‐lymphocyte ratio; SII, systemic immune‐inflammatory index; TLS, tertiary lymphoid structure.

^a^ Divided into SII high and SII low.

^b^ Divided into NLR high and NLR low.

^c^ Divided into PLR high and PLR low.

^d^ Divided into LMR high and LMR low.

### Comparison of nomogram diagnostic models

**FIGURE 5:**
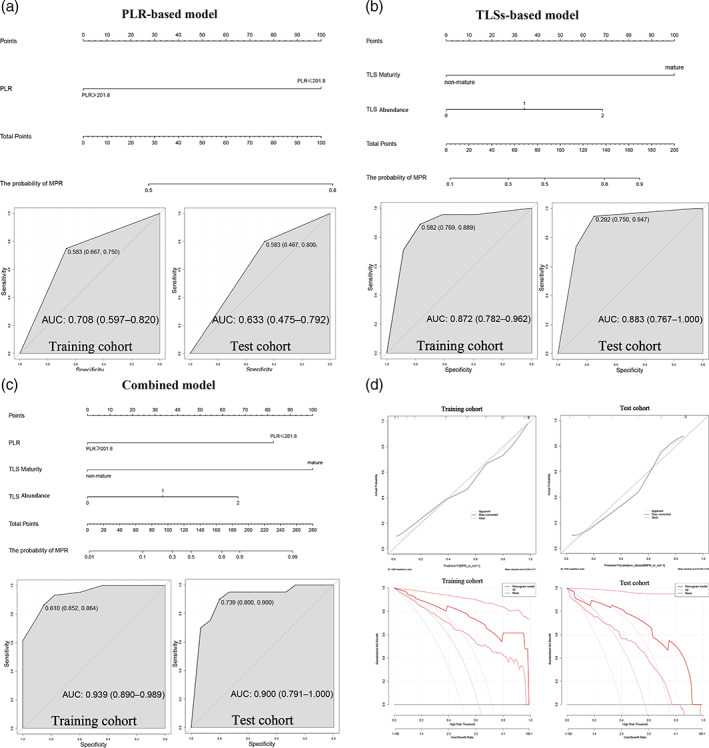
Combined model and ROC curves for the prediction of MPR in patients with NSCLC receiving neoadjuvant chemoimmunotherapy. (a) PLR‐based model and ROC curves for the prediction of MPR. (b) TLS‐based model and ROC curves for the prediction of MPR. (c) Combined model and ROC curves for the prediction of MPR. (d) Calibration curve close to ideal curve and DCA curve showing high clinical benefit and good model performance. DCA, decision curve analysis; MPR, major pathological response; NSCLC, non‐small‐cell lung cancer; PLR, platelet‐to‐lymphocyte ratio; ROC, receiver operating characteristic; TLS, tertiary lymphoid structure.

Nomogram models were constructed based on the PLR, TLS abundance, maturity, and their combination to predict patient MPR rates **(**Figure 5a–c **).** Comparison of area under the curve (AUC) values and the C‐index from ROC curves of the three models showed that for MPR prediction, the combined model (training set AUC = 0.939, validation set AUC = 0.900, C‐index = 0.939) was markedly superior to the PLR‐based model (training set AUC = 0.708, validation set AUC = 0.740, C‐index = 0.708) and TLS‐based model (training set AUC = 0.872, validation set AUC = 0.883, C‐index = 0.872) **(**Table 3 **)**. The calibration curve was close to the ideal curve, and the decision curve analysis showed high clinical benefit and good model performance (Figure 5d).

**TABLE 3:** Comparison of nomogram prognostic models.

End point	Models	AUC		C‐index
		Training cohort	Validation cohort	
The probability of MPR	Combined model	0.939	0.900	0.939
	PLR‐based model	0.708	0.740	0.708
	TLS‐based model	0.872	0.883	0.872

*Note*: Combined model: a combined nomogram model based on the expression of TLSs with PLR.

Abbreviation: AUC, area under curve; MPR, major pathological response; PLR, platelet‐to‐lymphocyte ratio; TLS, tertiary lymphoid structure.

## DISCUSSION

In this study, we investigated the relationship between pretreatment inflammatory biomarkers and postoperative tumor TLS status in 106 patients with NSCLC undergoing neoadjuvant immunochemotherapy. The results showed that the SII was negatively correlated with TLS abundance and maturity, and multivariate analysis confirmed that the SII was an independent risk factor influencing TLS abundance and maturity. Additionally, the PLR, TLS abundance, and maturity were independent predictors of the MPR. Notably, compared with using TLS features or PLR alone, combining both improved the AUC for predicting neoadjuvant treatment sensitivity to 0.939. These data indicate that the status of TLSs within the tumor microenvironment of patients with NSCLC may be modulated by systemic inflammatory processes. Furthermore, our results suggested that the integration of TLS metrics with inflammatory biomarkers may provide a more accurate diagnostic tool for predicting the efficacy of neoadjuvant immunotherapy combined with chemotherapy.

Systemic inflammation is an important manifestation of cancer progression. Muhammed et al. found that the NLR and PLR were associated with immunotherapy efficacy and were independent negative diagnostic factors in patients with HCC receiving ICI treatment.^24^ In immunotherapy for advanced NSCLC, increased NLR and PLR and poorer nutritional status are predictive of poorer overall survival.^25^ As immune cell aggregates, TLSs have been validated as immune predictors of the response to cancer immunotherapy, likely due to the induction of carcinoma‐associated fibroblasts, antigen presentation by dendritic cells, and anti‐tumor activities of CD8+ T and B cells within TLSs.^14^, ^17^, ^27^, ^28^ Zhou et al. found that TLS‐associated gene signatures could help stratify responders to immunotherapy in bladder cancer, providing strong evidence for the potential of TLSs to predict immunotherapy efficacy.^29^ As immune‐active regions in the tumor microenvironment, the status of TLSs is influenced by both local tumor factors and systemic immune conditions. TLS formation is induced by inflammatory microenvironmental factors and their abundance and structure undergo specific modulations based on inflammatory cues.^21^ Therefore, the combined assessment of TLSs and inflammatory cell counts representative of the inflammatory status enables a more comprehensive evaluation of an individual's immune response state. Additionally, reflecting TLS status changes through inflammatory biomarkers in the blood provides a preliminary basis for elucidating specific inflammatory microenvironments that promote TLS expression in NSCLC, while also offering new insights into immunomicroenvironment research.

This study is the first to investigate the factors that affect the formation of TLS in the tumor microenvironment from a unique perspective. The research discovered a negative correlation between TLS and the systemic inflammation indicator SII. This finding not only opened up new avenues for TLS research but also established SII as a reference indicator that reflects TLS status. This formed the foundation for investigating the relationship between TLS and the response to immunotherapy. More importantly, the predictive model that integrated TLS and PLR significantly improved the accuracy of assessing chemotherapy sensitivity in patients with NSCLC. This new approach will directly inform individualized treatment regimens and greatly advance the development of precision immuno‐oncology. In conclusion, this study pioneered a new perspective to elucidate the mechanisms of TLS formation and translated this discovery into improved clinical prediction of treatment sensitivity.

As a retrospective analysis, this study had inherent selection bias and limited robustness owing to its single‐center design. The limited amount of information on immunocombination chemotherapy in this study could have impacted the outcome of the analysis. Furthermore, there was a variation in tumor size and lymph node metastasis among the patients. The response of ≤10% residual tumor cells may have varied depending on the preimmunization tumor burden. Additionally, only a few inflammatory indices were examined, without comprehensive consideration of other potential influencing factors. Moreover, this study only included patients with resectable NSCLC receiving neoadjuvant immunochemotherapy, and chemotherapy effects cannot be excluded, precluding an accurate assessment of the relationship between PD‐1 inhibitors and TLSs. Future studies should validate the association between more extensive inflammatory biomarkers and TLSs through large‐scale prospective analyses, dynamically investigate the inflammatory factor‐TLS interplay, and experimentally explore the underlying molecular mechanisms. Finally, efforts to improve immunotherapy sensitivity by modulating the inflammatory status and extending research to other cancer types are warranted.

In conclusion, this retrospective study of 106 patients with NSCLC who received neoadjuvant immunochemotherapy, identified the SII, a systemic inflammation index, as an independent factor influencing TLS abundance and maturity, with a higher SII negatively associated with TLS expression in the tumor microenvironment. Moreover, compared with using TLSs or PLR individually, combining them more accurately predicted major pathological response rates to neoadjuvant immunochemotherapy.

## AUTHOR CONTRIBUTIONS

Study design: Shuanghu Yuan, Fuhao Xu; Data acquisition and analysis: He Zhu, Dali Xiong, Fuhao Xu; Interpretation of the data: Fuhao Xu, Kang Wang, Yinjun Dong, Li Li; Drafting of the manuscript: Fuhao Xu; Revision of the manuscript: Shuanghu Yuan. All authors contributed to the article and approved the submitted version.

## FUNDING INFORMATION

This study was supported in part by National Natural Science Foundation of China (grant no. NSFC82073345), Natural Science Foundation of Shandong Province (ZR202209010002).

## CONFLICT OF INTEREST STATEMENT

The authors declare that the research was conducted in the absence of any commercial or financial relationships that could be construed as a potential conflict of interest.

## Supporting information


**Supplementary Table S1.** The samples and clinical information of metagenomic sequencing included in the study.
**Supplementary Table S2.** The sample information of metagenomic sequencing.
**Supplementary Table S3.** The detection results of 48 cytokines.
**Supplementary Table S4.** List of pathogens detected in this study.
**Supplementary Table S5.** List of bacteria that were detected in all tumors.

**Supplementary Material 1.** Univariate and multivariate logistic analyses on inflammatory parameters and the expression of TLSs Density.
**Supplementary Material 2.** Univariate and multivariate logistic analyses on inflammatory parameters and the expression of TLSs Maturity.

## Data Availability

The datasets generated during and/or analyzed during the current study are available from the corresponding author on reasonable request.
