# Supplementary material for: Tertiary lymphoid structures combined with biomarkers of inflammation are associated with the efficacy of neoadjuvant immunochemotherapy in resectable non‐small cell lung cancer: A retrospective study
Source: Thorac Cancer. 2023 Dec 6;15(2):172–81. doi: 10.1111/1759-7714.15175 (PMC10788471; doi:10.1111/1759-7714.15175)
Supplement: Supplementary file 2 — Supplementary Material 1. Univariate and multivariate logistic analyses on inflammatory parameters and the expression of TLSs Density. Supplementary Material 2. Univariate and multivariate logistic analyses on inflammatory parameters and the expression of TLSs Maturity. [file TCA-15-172-s002.docx]

**SUPPLEMENTARY MATERIAL 1 | Univariate and multivariate logistic analyses on inflammatory parameters and the expression of TLSs Density**

| **Variable** | **Univariate analysis** | | **Multivariate analysis** | |
| --- | --- | --- | --- | --- |
|  | ***P*** | ***OR(95%CI)*** | ***P*** | ***OR(95%CI)*** |
|  |  |  |  |  |
| **Age** | 0.042 | 1.053(1.002-1.107) |  |  |
| **Gender** | 0.500 | 0.646(0.182-2.296) |  |  |
| male |  |  |  |  |
| female |  |  |  |  |
| **Smoking history** | 0.946 | 1.028(0.468-2.258) |  |  |
| smokers |  |  |  |  |
| never smokers |  |  |  |  |
| **Histology** | 0.348 | 0.660(0.277-1.572) |  |  |
| squamous carcinoma |  |  |  |  |
| adenocarcinoma |  |  |  |  |
| **TNM Stage** | 0.616 | 0.804(0.342-1.889) |  |  |
| Ⅰ+Ⅱ |  |  |  |  |
| Ⅲ |  |  |  |  |
| **SII^a^** | 0.001 | 0.258(0.114-0.584) | 0.017 | 0.273(0.094-0.796) |
| **NLR^b^** | 0.005 | 0.309(0.137-0.696) | 0.944 | 0.955(0.265-3.435) |
| **PLR^c^** | 0.105 | 0.517(0.233-1.148) |  |  |
| **LMR^d^** | 0.802 | 0.898(0.387-2.085) |  |  |
| **Lymphocyte count****^e^** | 0.024 | 2.722(1.142-6.488) | 0.206 | 2.142(0.658-6.974) |

CI=confidence interval. Statistical significance was set at P < 0.05. The expected count should be less than 5 to follow the Fisher's exact test results. ^a^Divided into SII high and SII low. ^b^Divided into NLR high and NLR low. ^c^Divided into PLR high and PLR low. ^d^Divided into LMR high and LMR low. **^e^** Divided into Lymphocyte count high and Lymphocyte count low.

**SUPPLEMENTARY MATERIAL 2 | Univariate and multivariate logistic analyses on inflammatory parameters and the expression of TLSs Maturity**

| **Variable** | **Univariate analysis** | | **Multivariate analysis** | |
| --- | --- | --- | --- | --- |
|  | ***P*** | ***OR(95%CI)*** | ***P*** | ***OR(95%CI)*** |
|  |  |  |  |  |
| **Age** | 0.993 | 1.000(0.955--1.046) |  |  |
| **Gender** | 0.309 | 0.491(0.125-1.933) |  |  |
| male |  |  |  |  |
| female |  |  |  |  |
| **Smoking history** | 0.361 | 0.685(0.305-1.542) |  |  |
| smokers |  |  |  |  |
| never smokers |  |  |  |  |
| **Histology** | 0.328 | 0.646(0.269-1.550) |  |  |
| squamous carcinoma |  |  |  |  |
| adenocarcinoma |  |  |  |  |
| **TNM Stage** | 0.032 | 0.352(0.135-0.916) | 0.061 | 0.376(0.135-1.048) |
| Ⅰ+Ⅱ |  |  |  |  |
| Ⅲ |  |  |  |  |
| **SII^a^** | ＜0.05 | 0.184(0.078-0.430) | 0.007 | 0.227(0.077-0.668) |
| **NLR^b^** | 0.007 | 0.323(0.143-0.731) | 0.611 | 0.753(0.252-2.246) |
| **PLR^c^** | 0.148 | 0.553(0.247-1.235) |  |  |
| **LMR^d^** | 0.118 | 1.960(0.843-4.558) |  |  |
| **Lymphocyte count^e^** | 0.075 | 2.766(0.903-8.468) |  |  |

CI=confidence interval. Statistical significance was set at P < 0.05. The expected count should be less than 5 to follow the Fisher's exact test results. ^a^Divided into SII high and SII low. ^b^Divided into NLR high and NLR low. ^c^Divided into PLR high and PLR low. ^d^Divided into LMR high and LMR low. **^e^** Divided into Lymphocyte count high and Lymphocyte count low.
